# Tracing the journey: Patient engagement in long-term antiretroviral therapy care in Malawi

**DOI:** 10.1371/journal.pone.0323983

**Published:** 2026-06-01

**Authors:** Hannock Tweya, Agness Thawani, Robin Klabbers, Jacqueline Huwa, Ethel Rambiki, Evelyn Viola, Layout Gabriel, Geldert Chiwaya, Joseph Chintedza, Aubrey Kudzala, Christine Kiruthu-Kamamia, Pachawo Bisani, Marrianne M. Holec, Caryl Feldacker

**Affiliations:** 1 Department of Global Health, University of Washington, Seattle, Washington, United States of America; 2 International Training and Education Center for Health (I-TECH), Seattle, Washington, United States of America; 3 Lighthouse Trust, Lilongwe, Malawi; 4 Department of Emergency Medicine, University of Washington, Seattle, Washington, United States of America; University of Dschang: Universite de Dschang, CAMEROON

## Abstract

**Background:**

Consistent engagement in antiretroviral therapy (ART) care is crucial to improve health outcomes and reduce HIV transmission. This study examined ART engagement patterns among ART clients during their first two years at two public ART clinics in Lilongwe, Malawi. Retention support for new initiates is provided by ART “Buddies” or through a two-way texting (2wT) system for those with phones and interest.

**Methods:**

ART engagement patterns were assessed over six-month intervals (>0–6, > 6–12, > 12–18, > 18–24) among clients stratified by retention support group: 1) Buddy without phone access; 2) Buddy with phone access; and 3) 2wT. Outcomes were based on ART program status at the end of each interval. Clients were classified as retained on ART or not retained (lost to follow-up (LTFU), transferred out, stopped, or died). Among those retained, engagement was further categorised as *continuously engaged* (attended all appointments within 13 days), *cyclically engaged* (returned late (14–59 days) at least once, or *re-engaged* (missed an appointment by ≥60 days but returned to care). Clients were not censored within interval and could re-enter care in subsequent intervals.

**Results:**

Among 6,303 clients, 5,880(93%) received ART Buddy support and 423(7%) received 2wT. Of those in the Buddy support group, 1,030 (18%) had phone access. 2wT clients showed the highest continuous engagement up to 18 months, compared to Buddy clients with and without phone access (70% vs 57% vs 30% at 0–6 months, 72% vs 58% vs 33% at >6–12 months, and 81% vs 76% vs 62% at >12–18 months). At 24 months, 3,363 (53%) were retained on ART: 2,790 (58%) of Buddy with phone; 277(27%) of Buddy without phone, and 296 (70%) of 2wT. Of those retained at 24 months, 1,834 (55%) were continuously engaged, 1,029 (31%) had cyclically engaged, and 500 (15%) re-engaged after LTFU across intervals.

**Conclusion:**

ART engagement was dynamic and heterogeneous over time. Tailored retention support based on engagement patterns and time on ART could improve long-term retention in ART care.

## Introduction

Retention in antiretroviral therapy (ART) care is essential for ensuring sustained viral suppression, improving health outcomes, and preventing the onward transmission of HIV [1–3]. ART retention, however, is challenging, particularly in low- and middle-income countries (LMIC) [4–7]. People living with HIV (PLHIV) in LMICs often face well-documented structural and individual barriers to HIV care, such as economic constraints, transportation difficulties, stigma, and mental health [8–10]. Previous studies reported that early disengagement, lack of retention within the first six months on ART, is particularly predictive of longer-term disengagement and loss to follow-up (LTFU) [11,12].

Traditionally, ART retention, defined as actively receiving ART, has been assessed cross-sectionally at 6, 12 or 24 months after ART initiation. At these time points, PLHIV are categorised as either ‘retained’ or ‘not retained’ [12–14]. This time-specific measure of ART retention has significantly shaped global policies but fails to capture the complexities of client behaviour between these time points. ART engagement is not uniform, but dynamic [15]. PLHIV frequently cycle in and out of care as they navigate changing personal, social, and economic circumstances [13]. ART engagement differs across times of ART follow-up and by client demographic groups [15,16]. Understanding ART engagement patterns - given that PLHIV frequently cycle in and out of care - is crucial for refining interventions and developing additional strategies to improve and sustain long-term retention. With the scale-up of electronic medical records systems (EMRS) which provide comprehensive, longitudinal data on PLHIV receiving ART, there is an opportunity to map client retention and ART engagement patterns in greater detail [12].

The Lighthouse Trust (LT) is a local non-governmental organisation providing HIV services and offers various support services to promote retention at its ART clinics, Lighthouse (LH) and Martin Preuss Centre (MPC), in Lilongwe, Malawi. These retention support services are provided by ART “Buddies”, two-way texting (2wT) and the Back-To-Care (B2C) program. ART Buddies are PLHIV who offer peer social support, encouragement, and reminders for upcoming and missed visits during the first 12 months of ART and are considered the standard of care (SoC) since 2019. 2wT is a hybrid intervention introduced in 2021 and combines an automated weekly blast of a non-HIV-related motivational message and specific, interactive, response-requested, ART visit reminders [17]. Preliminary findings demonstrated 2wT is effective, with a 91% ART retention rate at 12-months compared to 76% with SoC buddy support. Finally, B2C is a reactive ART retention support program offered to all clients as part of SoC (buddy and 2wT) in which clients who miss appointments by 14 days or more are traced by retention officers through phone and, if needed, home visit [18].

The objectives of this study were: 1) Describe characteristics of ART clients and ART program outcomes by type of retention support: Buddy (differentiating between clients with and without phone access) and 2wT; 2) Describe on-time clinic visit attendance over the first 24 months on ART; 3) Visualise ART engagement patterns over 24 months to understand how ART clients cycle in and out of care; and 4) Assess ART engagement patterns by sex, age group, and type of retention support at six-month intervals following ART initiation.

## Methods

### Study design

This retrospective descriptive cohort study used routine program data from the LT clinics (LH and MPC) in Lilongwe, Malawi. The study included all ART clients who initiated ART between 01 January 2020 and 31 December 2021, allowing a 24-month ART follow-up period by 31 December 2023.

### Settings

LT clinics use point-of-care (PoC) EMRS for client management [19]. Clients diagnosed with HIV are registered in the PoC EMRS and referred to an ART Buddy for pre-ART initiation counselling and psychosocial support. Clients are then assessed to determine their WHO HIV clinical stage. ART appointments are typically scheduled monthly for the first three months. After six months, appointments are scheduled every three or six months, depending on the client’s clinical condition. In addition to their standard ART supply, clients receive an additional two-days’ worth of ART for each appointment to prevent treatment interruptions. Clients transferring to LT clinics from other ART facilities are also registered in the EMRS and receive the same ART and adherence support. MoH ART outcomes are 1) Retained on ART, for clients who continue treatment; 2) LTFU, for clients who miss clinic appointments for at least 60 days; 3) Stopped ART treatment, for clients who discontinue treatment on their own volition or by ART provider’s decisions on account of suspected treatment failure and adverse events; 4) Transfer-out, for clients who moved their ART care to another ART facility; and 5) Died, for clients who have passed away due to any cause.

### ART retention support

Following pre-ART counselling, clients are offered the choice between early retention support by SoC Buddies or through the 2wT platform, as previously described [17]. Phone ownership and receipt of a confirmation registration message are a prerequisite for 2wT participation. Clients who opt for SoC Buddies receive phone calls reminding them of clinic appointments and in-person adherence counselling during clinic visits in the first 12 months of ART. Clients who opt for 2wT support receive personalised visit reminders on days 3 and 1 before appointments and on days 2, 5 or 11 after missed appointment visits, if applicable. Clients are asked to respond to visit reminders with “yes” or “no” to confirm attendance and may reschedule an appointment within 13 days. Additionally, the 2wT-supported clients receive weekly motivational messages on various health topics. Clients can interact with a 2wT officer via SMS during routine clinic hours. Both Buddy-supported and 2wT-supported clients who miss appointments for 14 days or more are followed up by the B2C team via phone or home visits. During the study, the 2wT retention support was offered for up to 24 months post-ART initiation.

### Study definitions

Clients were divided into 3 ART retention support groups: Buddy with phone (client had a recorded mobile phone number in the EMRs); Buddy without phone (client did not have a recorded mobile number in the EMRs), and 2wT (mobile phone required and used for 2wT enrolment).

We defined three outcome measures: On-time clinic visit attendance, retention and ART engagement patterns.

a On-time clinic visit attendance: A visit-level outcome defined as the proportion of clients with a scheduled ART refill appointment who attended the appointment within 13 days of the scheduled date. Clients often had multiple visits per 6-month interval.
b Overall retention was defined as the proportion of clients retained on ART at 24-month post-initiation among those who initiated ART between 01 January 2020 and 31 December 2021. Follow-up time during the first 24 months on ART was divided into six-month intervals (>0–6, > 6–12, > 12–18, and >18–24) to assess temporal patterns in engagement. Each six-month interval was treated as an independent observation period. Interval-specific retention was defined as the number of clients who received ART during a given interval and were retained on ART, divided by the number of clients who received ART during the same interval. Clients who transferred out, stopped ART treatment, or were LTFU were not censored within the interval and were eligible to re-enter the cohort in subsequent intervals if they returned to care.
c ART engagement patterns were assessed to deepen understanding of client behaviours of cycling in and out of care. ART engagement patterns were derived from client’s MoH ART program outcome at the end of each six-month interval and within-interval appointment attendance patterns. Clients who disengaged (MoH ART outcomes: LTFU, transferred out, stopped ART treatment, or died) from ART care at a facility at the end of any interval were assigned that outcome for that entire interval. Clients with the MoH ART outcome “retained on ART” at the end of any interval (consistent with the definition of retention above) were further categorised based on their within-interval appointment attendance pattern. These clients were considered: 1) Continuously engaged if they attended all appointments within 13 days of the scheduled date; 2) Cyclically engaged if they returned late (between 14 and 59 days) for at least one appointment in the interval; 3) Re-engaged, if they missed at least one appointment by 60 days or more in the interval but returned to care before the end of that six month interval. Clients who were LTFU, transferred out or stopped ART treatment were not censored at the end of the six-month interval to allow for potential returning to care.

In addition to assessing interval-level engagement, we determined an overall engagement pattern for each client over the full 24-month period using the same definitions. For instance, a client who attended all scheduled visits within 13 days of the scheduled date was considered “continuously engaged” throughout the first 24 months of care.

### Statistical analysis

Study data from the PoC EMRS at LH and MPC were extracted between 10 March and 20 May 2024. Data were analysed using Stata version 18. Descriptive statistics (frequencies and medians with interquartile range (IQR)) were used to describe the study population. On-time clinic visit attendance was assessed overall and monthly. ART retention and engagement pattern proportions were presented for each six-month interval, stratified by sex, age group, and type of retention support: Buddy support with or without phone and 2wT. We used Z-test for proportions and chi-square tests for trends to explore differences and trends in MoH ART outcomes, retention and ART engagement patterns across demographic and ART retention support groups. A 95% confidence interval and p-values were presented to aid interpretation of observed differences.

ART engagement patterns were visualised using a Sankey chart, illustrating trajectories for those retained on ART (continuously engaged, cyclically engaged, re-engaged) and those who disengaged (LTFU, stopped ART, transferred-out, died) at 6, 12, 18, and 24 months after ART initiation. To enhance visualisations simplicity and interpretability, two Sankey charts were created based on a qualitative segmentation approach: one for common patterns illustrated by ≥21 clients and another for rare patterns with 20 or fewer clients. Calculations of common pattern counts were based on the entire follow-up period for each client, including clients who disengaged 24 months before.

### Ethical considerations

Ethical approval was obtained from the Malawi National Health Sciences Research Committee (Protocol #23/10/4258). As this study used routine program data without client identifiers, participant informed consent was waived.

## Results

### Study population

A total of 6,990 PLHIV initiated ART between 1 January 2020 and 31 December 2021 at LH and MPC. Of them, 687 (10%) were excluded from analysis because they had received ART for six months or more at other sites prior to enrolment in care at LH and MPC. The remaining 6,303 (90%) were included: 1,030 (16%) in the Buddy support group with phone, 4,850 (77%) in the Buddy support group without phone, and 423 (7%) in the 2wT support group (phone required) (Table 1). Of those included, 58% were female and 42% male.

**Table 1:** Baseline characteristics and ART retention outcomes of PLHIV who received ARVs between 2020 and 2023 at Lighthouse and Martin Preuss Centre, Lilongwe, Malawi.

	Total	Retention Support Groups			
		Buddy support without phone	Buddy support with phone	Two-way Texting	Chi-square test^¥^
	6,303	1,030 (16%)	4,850 (77%)	423 (7%)	
Sex					<0.001
Female	3,640 (58%)	681 (66%)	2,735 (56%)	224 (53%)	
Male	2,663 (42%)	349 (34%)	2,115 (44%)	199 (47%)	
Age at ART initiation (years)					<0.001
18-34	3,437 (55%)	639 (62%)	2,574 (53%)	224 (53%)	
35-49	2,419 (38%)	325 (32%)	1,922 (40%)	172 (41%)	
50+	447 (7%)	66 (6%)	354 (7%)	27 (6%)	
WHO stage					0.206
1 or 2	4,812 (79%)	797 (81%)	3,695 (79%)	320 (76%)	
3	868 (14%)	133 (14%)	663 (14%)	72 (17%)	
4	394 (6%)	53 (5%)	313 (7%)	28 (7%)	
With ≥ 1 + scheduled clinic					<0.001
Yes	5,892 (93%)	922 (90%)	4,559 (94%)	411 (97%)	
No	412 (7%)	108 (10%)	291 (6%)	12 (3%)	
Overall on-time clinic attendance ^β^					<0.001
Yes	2,396 (41%)	198 (21%)	1,959 (43%)	239 (58%)	
No	3,496 (59%)	724 (79%)	2,600 (57%)	172 (42%)	
MoH ART outcomes at 24 months					<0.001
Retained on ART	3,363 (53%)	277 (27%)	2,790 (58%)	296 (70%)	
Disengaged	2,940 (47%)	753 (73%)	2,060(42%)	127(30%)	
LTFU	1,564 (25%)	457 (44%)	1,050 (22%)	57 (13%)	
Stopped treatment	290 (5%)	59 (6%)	219 (5%)	12 (3%)	
Transferred out	889 (14%)	209 (20%)	634 (13%)	46 (11%)	
Died	197 (3%)	28 (3%)	157 (3%)	12 (3%)	

Notes: ^¥^Chi-square test for association; MoH = Ministry of Health, WHO = World Health Organization, PLHIV = people living with HIV, ARVs = antiretrovirals; Having a phone was defined based on the availability of a recorded phone in the EMRs; ^β^Overall on-time attendance = Attended all visits within 13 days of the appointment.

Sex, age group at ART initiation, on-time clinic visit attendance and MoH ART outcomes at 24 months (χ^2^ test, p < 0.001) varied by retention support. More females than males did not have a recorded mobile phone number in the EMRS (female: 66% vs male: 34%, z-test: p < 0.001). Clients aged 18–34 years accounted for 62% of those in the Buddy support group without phone compared to those in the Buddy with phone (53%, z-test, p < 0.001) and the 2wT group (53%, z-test, p = 0.002).

### Overall on-time clinic visit attendance

Of the 6,303 PLHIV, 5,892 (93%) had at least one scheduled clinic visit with a median of 9 (IQR: 7–10) visits over 24 months (Table 1). Among those with scheduled clinic visits, 41% attended all appointments “on time”. On-time visit attendance was higher in the 2wT support group compared to the Buddy support group with phone (58% vs 43% (z-test, p < 0.001) and Buddy support group without phone (58% vs 21% (z-test, p < 0.001).

**Fig 1:**
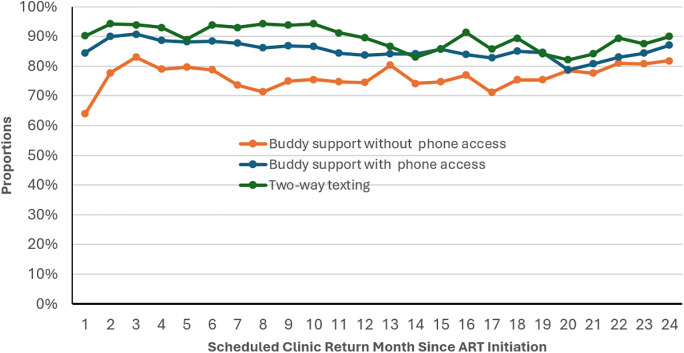
On-time clinic visit attendance among clients scheduled to return each month since ART initiation (2020 - 2023) at Lighthouse and Martin Preuss Centre, Lilongwe, Malawi by retention support group.

Monthly average on-time clinic visit attendance varied between 80% and 90%, with medians of 90% (IQR: 86–93) for the 2wT support group, 84% (IQR: 83–87) for the Buddy support group with phone and 76% (IQR: 74–79) for the Buddy support group without phone. (Fig 1)

### MoH ART outcomes and retention at 24-month post-ART initiation

At 24 months post-ART initiation, 3,363 (53%) were retained on ART and 2,940 (47%) disengaged from care: 1,564 (25%) were LTFU, 290 (5%) stopped ART treatment, 889 (14%) transferred out and 197 (3%) died (Table 1). Clients in the 2wT group had a higher 24-month ART retention compared to clients in the Buddy support group with phone (70% vs 58%, z-test: p < 0.001) and the Buddy support group without phone (70% vs 27%, z-test: p < 0.001).

### ART engagement patterns: Overall and by six-month intervals

Among the 3,363 clients retained on ART at 24 months and looking across interval engagement, 1,834 (55%) were continuously engaged, 1,029 (31%) were cyclically engaged, and 500 (15%) re-engaged. Only 1,834 (29% of the total cohort of 6,303) were continuously engaged in care across all four 6-month intervals over 24 months.

**Fig 2:**
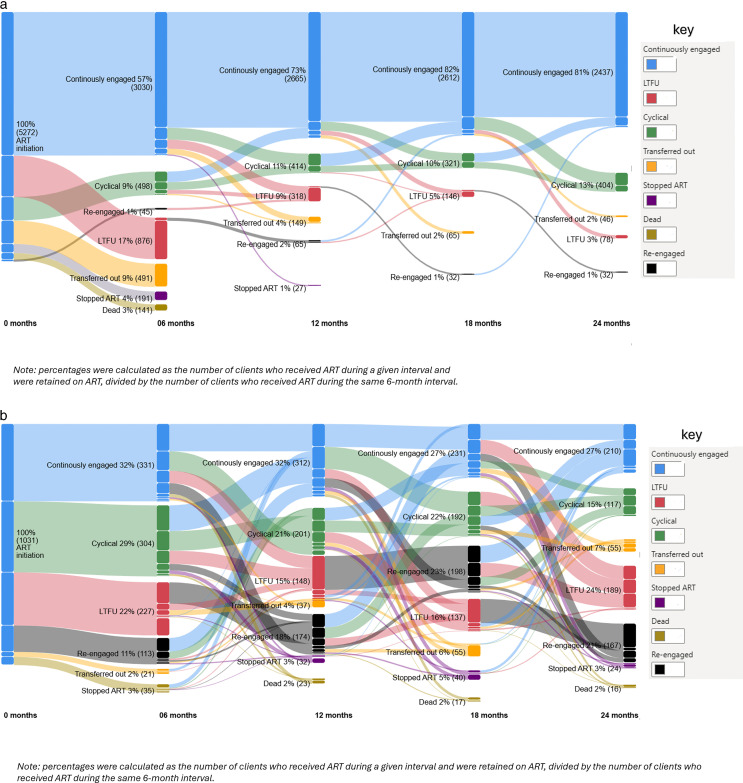
a. Common ART engagement patterns by six-month intervals during the first 24 months of ART. b. Rare ART engagement patterns by six-month intervals during the first 24 months of ART patterns.

Fig 2a and 2b Sankey charts visually summarise ART engagement over 24 months. The thickness of the lines represents the number of clients following each pathway, highlighting the relative frequency of different engagement trajectories. This visual approach also shows flow of clients between states, such as re-engagement after LTFU or transitions from cyclical to continuous engagement. Pattern counts (≥21 or <20) were engagement trajectories based on the entire follow-up period for each client, including clients who disengaged before 24 months.

Fig 2a shows ART engagement patterns trajectories by six-month interval in the first 24 months of ART, with each pattern representing at least 21 clients across their full follow-up. There were 33 common engagement patterns which accounted for 5,272 (84%) ART clients. In the first six months, 3,030 (57%) remained continuously engaged, 498 (9%) were cyclically engaged, 45 (1%) re-engaged, 876 (17%) were LTFU, 491 (9%) transferred out, 191 (4%) stopped ART and 141 (3%) died. Between 6 and 12 months, 2,665 (73%) were continuously engaged, with the proportion increasing to above 80% between 18 and 24 months. Notably, 45 (2%) clients who were LTFU within the first six months re-engaged in care before the end of the same six-month period. Some clients who were cyclically engaged at 12 months transitioned to continuous engagement at 18 or 24 months. Between 18 and 24 months, 81% of clients were continuously engaged.

Fig 2b shows engagement patterns representing 20 or fewer clients who were not included in Fig 2a. A total of 1031 (16%) ART clients had 276 engagement patterns trajectories between months 0 and 24, each occurring in fewer clients and representing greater variability than the patterns shown in Fig 2a.

#### ART engagement patterns by sex.

At 24 months, 1,458 (55%) males and 1,905 (52%) females were retained on ART (z-test, p < 0.001) (Table 2). In the first six months of ART, males were slightly more continuously engaged than females (80% vs 76% z-test, p < 0.001). After the first six months, levels of continuous engagement were comparable between males and females. The proportions of LTFU, cyclically engaged, and re-engagement after LTFU were similar by sex.

**Table 2:** Baseline characteristics and outcomes of PLHIV who received ART between 2020 and 2023 at Lighthouse and Martin Preuss Centre, Lilongwe, Malawi.

	Total	Sex^*¥*^	
Engagement patterns^‡^		Male	Female
**Total patients**	6,303 (100%)	2,663 (42%)	3,640 (58%)
**Overall: 0–24 months**			
Retained on ART	3,363 (53%)	1,458 (55%)	1,905 (52%)*
Continuously engaged	1,834 (55%)	816 (56%)	1,018 (53%)
Cyclically engaged	1,029 (31%)	435 (30%)	594 (31%)
Re-engaged	500 (15%)	207 (14%)	293 (15%)
LTFU	1,564 (25%)	644 (24%)	920 (25%)
Stopped ART	290 (5%)	128 (5%)	162 (4%)
Transferred out	889 (14%)	325 (12%)	564 (15%)*
Dead	197 (3%)	108 (4%)	89 (2%)
**0 to 6 months**			
Total patients	6,303	2,663	3,640
Retained on ART	4,321 (69%)	1,835 (69%)	2,486 (68%)
Continuously engaged	3,361 (78%)	1,462 (80%)	1,899 (76%)*
Cyclically engaged	802 (19%)	324 (18%)	478 (19%)
Re-engaged	158 (4%)	49 (3%)	109 (4%)
LTFU	1,103 (17%)	459 (17%)	644 (18%)
Stopped ART	226 (4%)	104 (4%)	122 (3%)
Transferred out	512 (8%)	187 (7%)	325 (9%)*
Dead	141 (2%)	78 (3%)	63 (2%)
**>6–12 months**			
Total patients	4,565	1,944	2,621
Retained on ART	3,831 (84%)	1,617 (83%)	2,214 (84%)
Continuously engaged	2,977 (78%)	1,261 (78%)	1,716 (78%)
Cyclically engaged	615 (16%)	250 (15%)	365 (16%)
Re-engaged	239 (6%)	106 (7%)	133 (6%)
LTFU	466 (10%)	216 (11%)	250 (10%)
Stopped ART	59 (1%)	32 (2%)	27 (1%)
Transferred out	186 (4%)	65 (3%)	121 (5%)
Dead	23 (1%)	14 (1%)	9 (0%)
**>12–18 months**			
Total patients	4,046	1,722	2,324
Retained on ART	3,586 (89%)	1,542 (90%)	2,044 (88%)
Continuously engaged	2,843 (79%)	1,204 (78%)	1,639 (80%)
Cyclically engaged	513 (14%)	228 (15%)	285 (14%)
Re-engaged	230 (6%)	110 (7%)	120 (6%)
LTFU	283 (7%)	117 (7%)	166 (7%)
Stopped ART	40 (1%)	15 (1%)	25 (1%)
Transferred out	120 (3%)	42 (2%)	78 (3%)
Dead	17 (0%)	6 (0%)	11 (0%)
**>18–24 months**			
**Total patients**	3,775	1,629	2,146
Retained on ART	3,367 (89%)	1,461 (90%)	1,906 (89%)
Continuously engaged	2,647 (79%)	1,160 (79%)	1,487 (78%)
Cyclically engaged	521 (15%)	216 (15%)	305 (16%)
Re-engaged	199 (6%)	85 (6%)	114 (6%)
LTFU	267 (7%)	108 (7%)	159 (7%)
Stopped ART	24 (1%)	9 (1%)	15 (1%)
Transferred out	101 (3%)	41 (3%)	60 (3%)
Dead	16 (0%)	10 (1%)	6 (0%)

*‡ All outcomes are assessed at the end of each six-month interval. Total patients refers to clients with at least one ART visit during the 6-month interval;* ^*¥*^Statistical analysis: Pairwise comparisons were conducted using 95% confidence intervals from Z-tests with, (*) indicates statistically significant results (p < 0.001).

#### ART engagement patterns by age group.

Overall, among clients retained on ART, clients aged 50 years and above had the highest proportion of continuously engaged clients (70%), with continuous engagement decreasing among clients aged 35–49 (58%) and 18–34 (49%) (χ^2^ test for trend, p < 0.001)(Table 3). Among all study clients, LTFU was the highest in the 18–34 age group (28%) compared to 21% in the 35–49 group and 19% in the 50 and above group (χ^2^ for trend, p < 0.001). Proportion of deaths was highest in the 50 + years group (9%) (χ^2^ for trend, p < 0.001).

**Table 3:** ART engagement patterns by age group: overall and at six-month intervals (2020 and 2023) at Lighthouse and Martin Preuss Centre, Lilongwe, Malawi.

		Age at ART initiation			
Engagement patterns^‡^		18	35	50	Chi-square test for trends
Total patients		3,437 (55%)	2,419 (38%)	447 (7%)	
Overall: 0–24 months					
Retained on ART		1,673 (49%)	1,429 (59%)	261 (58%)	<0.001
Continuously engaged		820 (49%)	830 (58%)	184 (70%)	<0.001
Cyclically engaged		525 (31%)	441 (31%)	63 (24%)	0.225
Re-engaged		328 (20%)	158 (11%)	14 (5%)	<0.001
LTFU		978 (28%)	499 (21%)	87 (19%)	<0.001
Stopped ART		198 (6%)	85 (4%)	7 (2%)	<0.001
Transferred out		524 (15%)	312 (13%)	53 (12%)	<0.013
Dead		64 (2%)	94 (4%)	39 (9%)	<0.001
**0 to 6 months**					
Total patients		3,437	2,419	447	
Retained on ART		2,275 (66%)	1,741 (72%)	305 (68%)	0.133
Continuously engaged		1,686 (74%)	1,410 (81%)	265 (87%)	<0.001
Cyclically engaged		488 (21%)	283 (16%)	31 (10%)	<0.001
Re-engaged		101 (4%)	48 (3%)	9 (3%)	0.032
LTFU		685 (20%)	351 (15%)	67 (15%)	<0.001
Stopped ART		152 (4%)	67 (3%)	7 (2%)	<0.001
Transferred out		282 (8%)	192 (8%)	38 (9%)	0.941
Dead		43 (1%)	68 (3%)	30 (7%)	<0.001
**>6–12 months**					
Total patients		2,442	1,813	310	
Retained on ART	1,972 (81%)		1,577 (87%)	282 (91%)	0.058
Continuously engaged		1,467 (74%)	1,271 (81%)	239 (85%)	<0.001
Cyclically engaged		338 (17%)	238 (15%)	39 (14%)	0.472
Re-engaged		167 (8%)	68 (4%)	4 (1%)	<0.001
LTFU		303 (12%)	145 (8%)	18 (6%)	<0.001
Stopped ART		43 (2%)	16 (1%)	0 (0%)	0.001
Transferred out		115 (5%)	64 (4%)	7 (2%)	0.014
Dead		9 (<1%)	11 (1%)	3 (1%)	0.112
**>12–18 months**					
Total patients		2,113	1,640	293	
Retained on ART		1,811 (86%)	1,500 (91%)	275 (94%)	0.150
Continuously engaged		1,381 (76%)	1,218 (81%)	244 (89%)	0.002
Cyclically engaged		279 (15%)	213 (14%)	21 (8%)	0.065
Re-engaged		151 (8%)	69 (5%)	10 (4%)	<0.001
LTFU		189 (9%)	85 (5%)	9 (3%)	<0.001
Stopped ART		27 (1%)	13 (1%)	0 (0%)	0.023
Transferred out		82 (4%)	35 (2%)	3 (1%)	<0.001
Dead		4 (<1%)	7 (<1%)	6 (2%)	<0.002
**>18–24 months**					
Total patients		1,929	1,567	279	
Retained on ART		1,674 (87%)	1,431 (91%)	262 (94%)	0.235
Continuously engaged		1,260 (75%)	1,151 (80%)	236 (90%)	0.002
Cyclically engaged		282 (17%)	217 (15%)	22 (8%)	0.033
Re-engaged		132 (8%)	63 (4%)	4 (2%)	<0.001
LTFU		166 (9%)	89 (6%)	12 (4%)	<0.001
Stopped ART		16 (1%)	8 (1%)	0 (0%)	0.074
Transferred out		65 (3%)	31 (2%)	5 (2%)	0.013
Dead		8 (<1%)	8 (1%)	0 (0%)	<0.001

‡ All outcomes are assessed at the end of each six-month interval. Total patients refers to clients with at least one ART visit during the 6-month interval.

In the six-month interval analysis, clients aged 50 years and above had the highest proportion of continous engagement among clients retainedon ART, with 87% at 0–6 months, 85% at >6–12 months, 89% at >12–18 months, and 94% at >18–24 months, compared to 81%,81%, 81%, 80% in ages 35–49 years and 74%, 74%, 76% and 75% in ages 18–34 years, respectively (χ^2^ for trend, all p-values <0.001). Cyclical engagement was generally lower in ages 50 years and above, ranging between 8% and 14%, while in other younger age groups, it was consistently ≥14%.

#### ART engagement patterns by type of ART retention support.

ART engagement patterns varied by type of retention support in all six-month intervals (Table 4). Sixty-eight percent of clients in the 2wT group were continuously engaged compared with 55% in the Buddy group with phones and 36% in the Buddy group without phones (z-test, p < 0.001).

**Table 4:** ART engagement patterns by ART retention group: overall and by six-month intervals (2020–2023) at Lighthouse and Martin Preuss Centre, Lilongwe, Malawi.

Engagement patterns^‡^	ART Retention Support Groups			
	Buddy support without phone access^β^	Buddy support with phone access	Two-way texting	Chi-square test^¥^
Total patients	1,030 (16%)	4,850 (77%)	423 (7%)	
**Overall: 0–24 months**				
Retained on ART	277 (27%)	2,790 (58%)**	296 (70%)**	<0.001
Continuously engaged	100 (36%)	1,532 (55%)**	202 (68%)**	
Cyclically engaged	114 (41%)	857 (31%)	58 (20%)	
Re-engaged	63 (23%)	401 (14%)	36 (12%)	
LTFU	457 (44%)	1,050 (22%)**	57 (13%)**	
Stopped ART	59 (6%)	219 (5%)	12 (3%)	
Transferred out	209 (20%)	634 (13%)**	46 (11%)	
Dead	28 (3%)	157 (3%)	12 (3%)	
**0 to 6 months**				
Total patients	1,030	4,850	423	
Retained on ART	478 (46%)	3,490 (72%)**	353 (83%)**	<0.001
Continuously engaged	314 (66%)	2,751 (79%)**	296 (84%)**	
Cyclically engaged	120 (25%)	632 (18%)	50 (14%)	
Re-engaged	44 (9%)	107 (3%)	7 (2%)	
LTFU	346 (34%)	722 (15%)**	35 (8%)**	
Stopped ART	50 (5%)	171 (4%)	5 (1%)**	
Transferred out	135 (13%)	356 (7%)	21 (5%)	
Dead	21 (2%)	111 (2%)	9 (2%)	
**>6–12 months**				
Total patients	537	3,663	365	
Retained on ART	366 (68%)	3,131 (85%)**	334 (92%)**	<0.001
Continuously engaged	228 (62%)	2,458 (79%)**	291 (87%)**	
Cyclically engaged	86 (23%)	498 (16%)	31 (9%)	
Re-engaged	52 (14%)	175 (6%)	12 (4%)	
LTFU	114 (21%)	337 (9%)**	15 (4%)	
Stopped ART	12 (2%)	43 (1%)	4 (1%)	
Transferred out	43 (8%)	132 (4%)	11 (3%)	
Dead	2 (0%)	20 (1%)	1 (0%)	
**>12–18 months**				
Total patients	397	3,304	345	
Retained on ART	318 (80%)	2,955 (89%)**	313 (91%)	<0.001
Continuously engaged	218 (69%)	2,355 (80%)**	270 (86%)*	
Cyclically engaged	72 (23%)	413 (14%)	28 (9%)	
Re-engaged	28 (9%)	187 (6%)	15 (5%)	
LTFU	53 (13%)	208 (6%)	22 (6%)	
Stopped ART	4 (1%)	33 (1%)	3 (1%)	
Transferred out	19 (5%)	95 (3%)	6 (2%)	
Dead	3 (1%)	13 (0%)	1 (0%)	
**>18–24 months**				
Total patients	344	3,107	324	
Retained on ART	278 (81%)	2,793 (90%)**	296 (91%)	<0.001
Continuously engaged	197 (71%)	2,205 (79%)**	245 (83%)	
Cyclically engaged	54 (19%)	430 (15%)	37 (12%)	
Re-engaged	27 (10%)	158 (6%)	14 (5%)	
LTFU	43 (12%)	208 (7%)	16 (5%)	
Stopped ART	4 (1%)	19 (1%)	1 (0%)	
Transferred out	17 (5%)	74 (2%)	10 (3%)	
Dead	2 (1%)	13 (0%)	1 (0%)	

βBuddy support with phone access: Defined as having a phone number recorded in the Electronic Medical Records (EMR) system. ^‡^All outcomes are assessed at the end of each six-month interval. Total patients refers to clients with at least one ART visit during the interval. ^¥^Statistical analysis: χ² tests were used to examine associations between types of retention support; Pairwise comparisons were conducted using 95% confidence intervals from Z-tests with, (*) indicates statistically significant results. Comparison groups: Buddy support without phone access vs. buddy support with phone access; Buddy support with phone access vs. two-way texting support. Denominator (total patients) is the number of clients who received ART during a specific six-month interval.

In the six-month interval analysis, the 2wT support group had the highest proportion of clients who continuously engaged up to 18 months post-ART initiation (84% at 0–6 months, 87% at >6–12 months, and 86% at >12–18 months), compared to the Buddy support group with phone (79%, 79%,80%) and without phone (66%, 62%,69%) (z-test, all p-values <0.02). Clients in the Buddy support group without phones consistently had the highest proportion of LTFU of 34% at 0–6 months and 21% at >6–12 months, as compared to LTFU rates in those same two periods in the Buddy support group with phones (15% and 9%) and 2wT group (8% to 4%) (z-test, p-values <0.001).

## Discussion

Understanding ART engagement patterns among PLHIV is vital for viral load suppression and resource allocation. This study describes demographics, on-time clinic visit attendance, retention and ART engagement patterns during the first 24 months of ART among clients receiving ART at two large, public clinics in Malawi. Overall, 53% of clients were retained at 24 months post-ART initiation, with the highest retention observed in the 2wT support group (70%) compared to those in the Buddy support group with phone (58%) and without phone (27%). Only 41% attended all clinic visits on time, with the lowest attendance among those without phones (21%). Of all clients, 47% disengaged from care (became LTFU, stopped ART treatment, transferred out and died), with half of them being LTFU. Most clients (84%) followed a limited set of common engagement patterns, while 16% had more varied, infrequently observed patterns. Only 29% of the total cohort remained continuously engaged throughout the 24-month period. Clients aged 50 + had the highest proportion of continuous engagement. Cyclically engaged was most common among those without a phone. We discuss several implications of these findings on practice and policy.

First, this study highlights the complexity of clients’ ART engagement behaviours, with significant variability over the first two years of ART. Engagement patterns varied across six-month intervals. While most clients (84%) demonstrated common engagement trajectories with minimal variability over time, a smaller group (16%) had varied and infrequent patterns. These findings highlight the potential for a differentiated approach to retention interventions, tailoring support intensity based on clients’ evolving needs. During the early phase of ART (0–6 months), a universal intensive support package – including enhanced counselling, Buddy or 2wT support – can be implemented, as early retention is critical for long-term ART adherence. Beyond six months, once client’s engagement patterns become apparent, a stratified strategy to retention support could be considered. Shifting from a uniform to a dynamic, data-informed retention approach has the potential to improve efficiency and retention outcomes; however further research is needed to evaluate the effectiveness and feasibility of such approaches.

Second, our study suggests that clients with phones had better ART engagement and on-time clinic visit attendance than clients without phones. 2wT participants received motivational messages, two visit reminder messages before each visit, and up to three additional messages if they missed an appointment [17]. Likewise, although not all ART buddies were able to remind clients of upcoming visits, ART retention buddies contacted their clients after missed visits. Regardless of contact method, clients with phone access to 2wT or buddies had higher engagement and more timely clinic visits, likely reflecting early identification of retention problems and swifter response to follow-up efforts. The observed disparity in retention by phone status may also be associated with socioeconomic and psychosocial differences between the retention groups. ART clients with phones tend to have higher education levels, greater economic ability and better health literacy [20–22], all of which can improve the ability to engage with healthcare services [23]. Additionally, those who provided their phone numbers may also be more likely to have disclosed their status while those without phone numbers may face more stigma or fear violence.

Third, demographics still matter. Older ART clients showed more stable engagement compared to younger clients, a finding consistent with other studies [24]. Although the reasons are not fully understood, higher retention in older adults could reflect established routines, fewer competing priorities, and stronger health-seeking behaviours. These findings highlight the importance of age-tailored interventions to improve engagement in younger populations. Also, as there are more female clients, yet more men than women have phones, both proactive and reactive retention efforts must address the persistent gender dimension to engagement and retention in care.

Fourth, while cyclically engaged or re-engaged patterns are associated with worse clinical outcomes, including risks of treatment failure and unsuppressed viral loads [25], not all cyclical engagement and re-engagement instances result from treatment interruptions. Some clients may obtain ART from other facilities while they travel or temporarily relocate for work [26], underscoring the need for improved communication with clients, better documentation of emergency ART provision and simplified transfer processes between clinics.

Lastly, as suggested by previous studies [11,15], the interval-based engagement indicators capture ART continuity more effectively than standard fixed-point retention measures. Unlike traditional program retention indicators, which only measure whether clients are in care at a single point in time, this approach identifies patterns of continuous engagement, cyclical attendance, and re-engagement after gaps in care. All clients who return to care are included in the interval in which they receive ART, ensuring that no participants are excluded from the analysis. This approach is particularly relevant for monitoring progress toward the 95-95-95 targets, as sustained engagement and continuity of care are essential for achieving viral suppression. Incorporating interval-specific engagement indicators into routine program monitoring, especially where EMR systems are available, can provide a more accurate picture of client adherence, identify periods of potential disengagement, and support targeted interventions to maintain long-term ART continuity.

### Strengths and limitations

This study is among the few to describe retention as a dynamic and evolving ART engagement, reasserting that client retention is not a binary state but a complex process. Differentiating client engagement patterns by type of retention support and phone access contributes to novel insights that can inform future retention service delivery strategies. Our study also had limitations. First, using a phone number as a proxy for phone access can be problematic for several reasons. Although phone numbers were confirmed at 2wT enrollment, some became inactive or did not work consistently throughout the study. Similarly, clients in SoC buddy support may have changed numbers during the study, and those categorised as without phones at enrolment may have obtained a phone later or may have had phone access later but did not share a number due to privacy concerns. These phone-based factors could have caused errors in client categorisation, potentially affecting observed ART engagement patterns. Second, clients were limited to those receiving care at two large urban sites, so observed cyclical and re-engagement patterns may not be representative of those found in smaller, rural or peri-urban clinics. Third, while utilising a large study population offers several advantages, some statistically significant differences of less than 5% between groups may not be meaningful for practice or policy change. Lastly, appointment rescheduling of fewer than 13 days was not accounted for in the classification of engagement patterns. This may have led to misclassification of some continuously engaged clients as having cyclical engagement patterns.

## Conclusion

ART engagement among study clients was dynamic and heterogeneous over time. The majority of ART clients followed a consistent and stable retention trajectory, with minimal variability. Engagement patterns varied by retention support type: clients who opted into 2wT showed higher levels of continuous engagement across retention periods. Strikingly, the finding that only 29% of clients were continuously engaged during the first 24 months underscores a substantial gap in continuous ART retention. These findings support the urgent need for further research to better identify clients at higher risk of disengagement and to explore tailored retention strategies that best match clients’ changing needs over time in ART care.
